# Iron Encapsulation by Deacetylated Glucomannan as an Excipient Using the Gelation Method: Characteristics and Controlled Release

**DOI:** 10.17113/ftb.60.01.22.7130

**Published:** 2022-03

**Authors:** Dyah H. Wardhani, Fatiha N. Etnanta, Hana N. Ulya, Nita Aryanti

**Affiliations:** 1 Department of Chemical Engineering, Faculty of Engineering, Universitas Diponegoro, Jl. Prof. Sudarto, SH, Tembalang, 50239 Semarang, Indonesia; 2 Institute of Food and Remedies Biomaterial (INFARMA), Universitas Diponegoro, Jl. Prof. Sudarto, SH, Tembalang, 50239 Semarang, Indonesia

**Keywords:** glucomannan deacetylation, gelation method, iron encapsulation

## Abstract

**Research background:**

Deacetylation and the use of CaCl_2_ as a gelation agent improve the performance of glucomannan as iron encapsulant. This study was conducted to investigate the effects of deacetylation degree and pH of gelation on the characteristics of encapsulated iron using gelation in CaCl_2_ solution.

**Experimental approach:**

Glucomannan was deacetylated at various NaOH concentrations and was subsequently utilized as an iron excipient using the pipette-dropped gelation method in CaCl_2_ solution to directly investigate the process of encapsulation by gelation. The pH of the gelation solution was also changed. The beads were subsequently vacuum-dried.

**Results and conclusions:**

Deacetylation led to lower endothermic peak of the glucomannan than that of the native one. The deacetylation degree and pH of gelation did not significantly affect the diameter of the beads but influenced their appearance and physical characteristics. The backbone of glucomannan was not changed by either the deacetylation degree or the pH of the gelation. The highest encapsulation efficiency (73.27%) was observed in the encapsulated iron using the glucomannan matrix of the highest deacetylation degree (82.56%) and gelated in the solution at pH=10. The highest deacetylation degree of glucomannan caused the highest swelling of the beads, which led to the release of a higher amount of iron. Glucomannan deacetylation improves the iron encapsulation and enables higher iron release at pH=6.8 than at pH=1.2. The Weibull model was the best-fitting model to represent the profile of iron release from the deacetylated glucomannan matrix using the gelation method (R^2^>0.93) at pH=6.8 and pH=1.2.

**Novelty and scientific contribution:**

This result supports the application of deacetylated glucomannan using NaOH as a pH-sensitive matrix for iron encapsulation and CaCl_2_ solution as gelation agent. A higher deacetylation degree leads to the release of a higher amount of iron from the matrix. The encapsulation does not only protect the iron but also delivers it to the absorption site and controls its release, which is useful in supplement formulation or food fortification. The results show that the deacetylated glucomannan as the matrix holds more iron in encapsulation process.

## INTRODUCTION

Iron is an essential nutrient that supports various metabolic activities, including oxygen transfer, DNA formation, immune system improvements and nitric oxide metabolism (*1*). More than 1.2 billion people worldwide are affected by iron-deficiency anaemia (*2*). Despite the abundance of iron sources in nature, the iron levels in the human body are controlled only by absorption (*3*). Iron absorption in the body is affected by many factors, including the form of iron compound. Iron is easily oxidized due to environmental changes (*4*). Oxidized iron is less soluble and is unavailable for absorption in the human gastrointestinal tract (*3*); hence, non-oxidized iron needs to be protected (*5*). A protective barrier to retain the active state of certain ingredients using an encapsulation method has been previously reported (*6*). Aside from covering and protecting the active compound, a suitable encapsulant can also be used to control its release during the absorption in the gastrointestinal tract. Absorption of most dietary iron occurs in the duodenum and proximal jejunum (*3*). The pH of the gastrointestinal tract is rapidly changed from highly acidic in the stomach to about pH=6 in the duodenum (*7*).

Polysaccharides exhibit excellent capabilities as encapsulants for reducing the iron oxidation and maintaining the iron nutritional value (*8*). Glucomannan, one of the highly viscous polysaccharides, is composed of mannose and glucose linked by β-1-4 glycosidic bonds with acetyl groups attached to the saccharide units (*7*). Ulya *et al.* (*9*) and Wardhani *et al.* (*10*) reported that the release of glucomannan-based encapsulant was higher at pH=6.8 than at pH=1.2. This property supports the application of glucomannan for the controlled release of iron. Hydrolyzed glucomannan reduces viscosity and successfully protects iron when using the spray-drying encapsulation method (*8*). However, encapsulation using the gelation method requires different glucomannan modifications to maximize iron protection (*11*).

Glucomannan is widely used as a food material owing to its nontoxic properties and ability to produce thermostable gel (*12*). Removing the acetyl groups of the polysaccharides, known as deacetylation, regulates hydrogen and hydrophobic bonds that induce gelling properties (*11*–*13*). It has been reported that deacetylation using alkali induced gelation in glucomannan (*14*). Deacetylated glucomannan was found to be more firm, elastic and stable than the native one at low pH and high temperature (*15*). These properties are important to support its application, such as in films (*16*), restructured seafood products (*13*), and encapsulation using the gelation method (*9*, *10*, *17*, *18*). Deacetylation has been reported as a method for improving the loading efficiency of vitamin C (*10*) and controlling the drug release from the chitosan matrix (*19*).

The effects of KOH, NaOH and Na_2_CO_3_ under either homogeneous or heterogeneous reaction on the extension of the deacetylated glucomannan gelation have been studied (*12*, *17*, *20*, *21*). NaOH exhibited a more significant effect on glucomannan deacetylation than KOH owing to its stronger ionization ability (*22*). Moreover, Ulya *et al.* (*9*) reported that the encapsulation efficiency of deacetylated glucomannan in encapsulating iron using ethanol as gelation medium was just above 60%. Gelation using ethanol resulted in the formation of a film-like layer instead of beads as the encapsulation product, which facilitated the loss of iron during gelation (*18*). Wardhani *et al.* (*10*) successfully encapsulated vitamin C with the CaCl_2_ solution as a gelation agent, and deacetylated glucomannan to form beads with an encapsulation efficiency of 85%.

Although many studies of glucomannan deacetylation have been performed, a comprehensive study using NaOH for iron encapsulation and CaCl_2_ as the gelation agent has not yet been explored. The combination of NaOH and CaCl_2_ was superior to that of NaOH and ethanol for the iron encapsulation by gelation (*18*). However, such a condition has not been further studied. Hence, the objective of this study is to investigate the effect of the deacetylation degree and pH of gelation on the characteristics of encapsulated iron using the CaCl_2_ gelation method. This modification and encapsulation will offer new insights into the application of glucomannan.

## MATERIALS AND METHODS

### Materials

Food-grade glucomannan from *Amorphophallus oncophyllus* flour (purity 91%) was purchased from a local seller in Nganjuk, East Java, Indonesia. Iron(II) sulfate heptahydrate, NaOH, KOH, HCl, CaCl_2_, 1,10-phenanthroline and other chemicals of analytical grade were purchased from Merck Chemical Co. (Darmstadt, Germany). Phosphate-buffered solution (pH=6.8) was prepared by dissolving Na_2_HPO_4_ and NaH_2_PO_4_ in distilled water (Merck Chemical Co.). Ethanol (96%) was obtained from PT. Indo Acidatama (Surakarta, Indonesia).

### Deacetylation of glucomannan

With slight modifications, glucomannan was deacetylated using the method described by Wardhani *et al.* (*21*). Glucomannan (1 g) was deacetylated in 100 mL of various concentrations of NaOH solution (8–32 g/L) for 1 h under constant stirring. Deacetylation was stopped by neutralizing the pH of the solution using a sulfuric acid solution (0.1 M). Then, the deacetylation degree of each deacetylated glucomannan solution was determined.

### Iron encapsulation: bead formation and bead diameter determination

The iron beads were prepared based on the procedure used by Wang and He (*23*). An FeSO_4_·7H_2_O solution (20 mL, 0.175 g/L) at about the concentration of total absolute iron requirement for adult female (*24*) was added to the deacetylated glucomannan solution (100 mL) under constant stirring for 30 min. The amount of FeSO_4_·7H_2_O was selected based on the common iron concentration in iron supplements. This solution was pipette-dropped into 150 mL of 29.4 g/L CaCl_2_ solution (pH=5.0). After 30 min, the beads were collected and the excess liquid was wiped. The diameter of the fresh beads was determined before they were placed in a desiccator for vacuum-drying. The diameter of the bead was determined using a Vernier caliper by averaging the diameter of five fresh beads.

### Determination of the degree of deacetylation

The degree of deacetylation (DD) was determined using the titration method (*25*). A suspension of the deacetylated glucomannan (1%, *m*/*V*) was prepared in 6 mL of 75% ethanol at 50 °C. After stirring for 30 min, the KOH solution (3 mL, 28.05 g/L) was added to the suspension and left to react for 24 h. This suspension was back-titrated with 3.65 g/L of HCl using phenolphthalein as an indicator. The DD was calculated as follows:



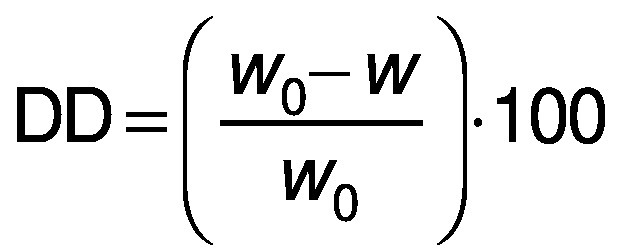



where *w*_0_ and *w* denote the acetyl mass fractions of native and deacetylated glucomannan, respectively. A blank without glucomannan addition was titrated in parallel. The acetyl content was calculated as follows:



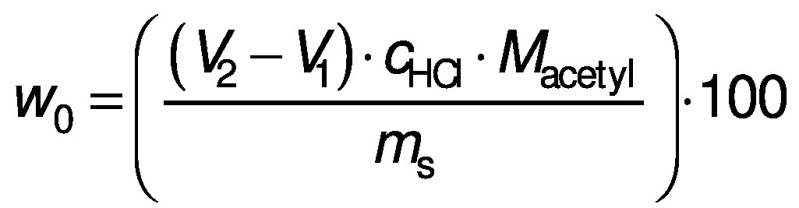



where (*V*_2_-*V*_1_) is the difference in the HCl volume in litre when titrating between the sample and the blank titrant, *c*_HCl_ is the concentration of the titrant, *M*_acetyl_ is the molecular mass of the acetyl group (43 g/mol), and *m*_s_ is the sample mass (g).

### Solubility and swelling analyses

Solubility and swelling analyses of dried iron beads were conducted at pH=1.2 and pH=6.8 of the solutions based on the modified method of Wang *et al.* (*26*). The beads (0.1 g) were immersed in 10 mL of either HCl solution (pH=1.2) or phosphate-buffered solution (pH=6.8), which was then heated to 60 °C for 30 min to accelerate the dissolution process. The supernatant was separated by centrifugation at 2300×*g* (EBA 21 centrifuge; Hettich®, Kirchlengern, North Rhine-Westphalia, Germany) for 20 min. These supernatants were decanted to aluminium vessels, which were then weighed before and after drying at 105 °C. The solubility and swelling of deacetylated glucomannan were calculated using the following equations, respectively:



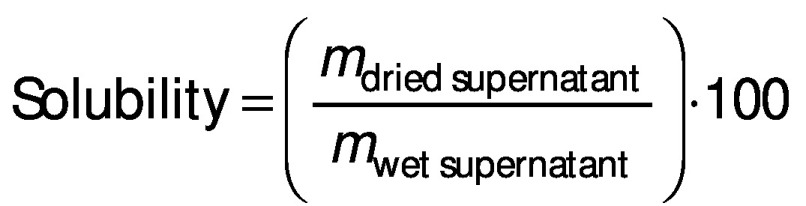





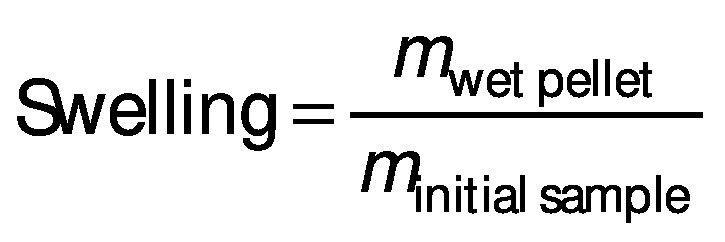



### Encapsulation efficiency

The iron concentration was measured to find the encapsulation efficiency using an ultraviolet-visible spectrophotometer (UV Mini 1240; Shimadzu Corp., Kyoto, Japan) at a wavelength of 510 nm. Dried iron beads (0.1 g) were placed in the flask together with the phenanthroline solution (10 mL, 1 g/L), sodium acetate buffer (8 mL, 98.4 g/L), and hydroxylamine hydrochloride solution (1 mL, 100 g/L). The solution was diluted to 50 mL using distilled water. After 15 min of stirring, the solution was filtered using Whatman filter paper (no. 41), and the absorbance of the filtrate was read at 510 nm. The concentration of the iron was determined by comparing it with the iron standard curve (0.35–122.5 mg/L). The encapsulation efficiency (EE) was further calculated using the following equation:



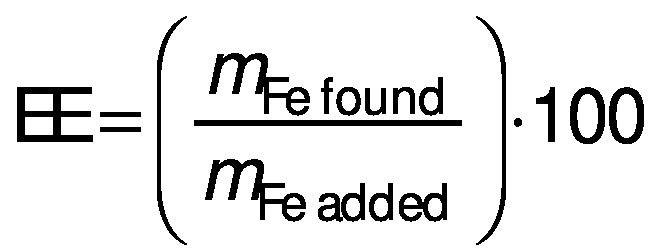



where *m*_Fe found_ and *m*_Fe added_ represent the mass of iron in the beads after encapsulation and the initial mass of iron, respectively.

### Functional groups, morphology and thermal determination

The functional groups and morphology of the dried beads of encapsulated iron samples (DD=82.56%, pH=10, DD=82.56%, pH=5 and DD=72.67%, pH=5) and native glucomannan were analyzed using the FT-IR Spotlight 200i instrument (PerkinElmer, Waltham, MA, USA) within a wavenumber range of 4000–400 cm^−1^ and scanning electron microscopy coupled with energy-dispersive X-ray (JEOL-JSM 6510LA; JEOL Ltd., Tokyo, Japan) at 2500× magnification. The thermal properties of the samples were analyzed using a Shimadzu DSC-60Plus differential scanning calorimeter (DSC; Shimadzu Corp., Kyoto, Japan) at 0–600 °C. A sample (3.2 mg) was placed in the aluminium crimp cell at a heating rate of 10 °C/min under an air atmosphere and a flow rate of 10 mL/min.

### Iron release and its kinetic model

Dried beads (0.1 g) were dissolved in two pH solutions (*i.e.* pH=1.2 and pH=6.8) and incubated at 37 °C. After 0–120 min, the iron release was determined. The iron release profile was studied using three mathematical models: the Korsmeyer–Peppas, Weibull, and Higuchi equations. The best-fitting model was revealed by the coefficient of determination (R^2^) and the root mean square error (RMSE), which was calculated using a linear regression method.

The equation of the Korsmeyer–Peppas model (*27*) is as follows:



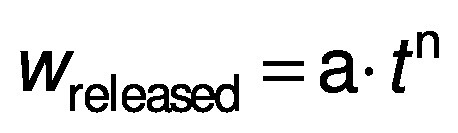



The released fraction of iron after the duration *t* (h) from the glucomannan matrix is represented by *w*_released_ (g/g) value, whereas a and n are the constants of the iron release rate and the iron release mechanism, respectively.

The Weibull model (*28*) is given here:



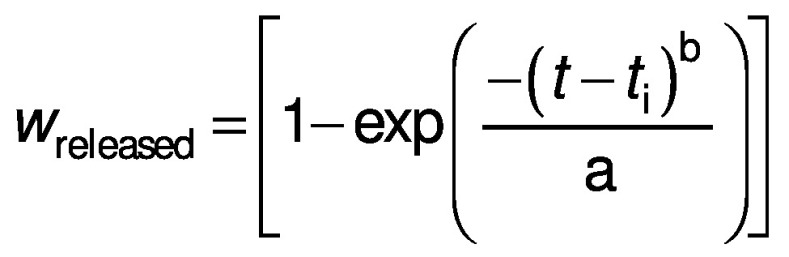



where *w*_released_ is the fraction of dissolved iron concentration in the solution after time *t*, *t*_i_ is the lag time needed for the iron release, a acts as a parameter depending on the time, and b describes the alteration shape of the dissolution curve. In this study, no lag time was observed, so it is assumed that the value of *t*_i_ is zero.

The Higuchi model is (*29*) given here:



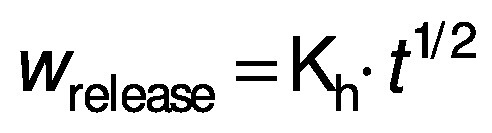



where K_h_ is the Higuchi constant and *t* is time of release.

### Statistical analysis

The data were presented as mean value±standard deviation of triplicate measurements. One-way analysis of variance was employed to evaluate the significant differences between sample mean values, with significant level considered at p<0.05 using the Statistical Package for the Social Sciences (SPSS) (*30*) software, v. 16.0 for Windows.

## RESULTS AND DISCUSSION

Glucomannan with the degrees of deacetylation (DD) ranging from 63.37 to 82.56% was prepared using different concentrations of NaOH. The deacetylated glucomannan was subsequently applied as an iron encapsulant by dropping it into CaCl_2_ solution for gelation to form beads followed by drying. The effects of the pH of the gelation solution on the properties of dried encapsulations were also determined.

### Thermal properties

**Fig. 1:**
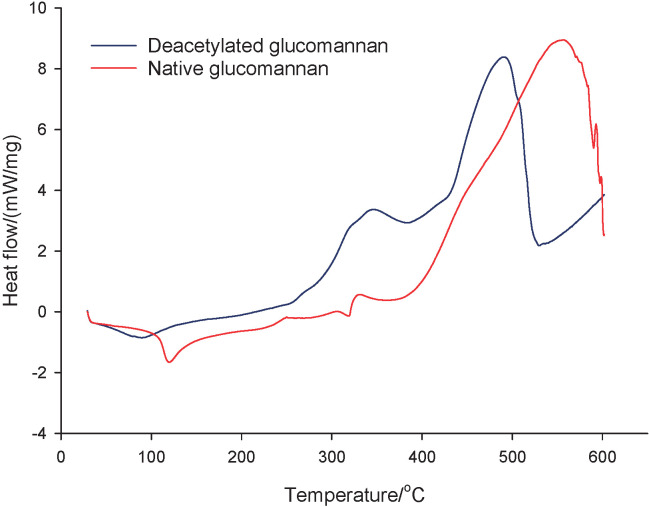
Differential scanning calorimetry curve of native and deacetylated glucomannan obtained at 82.58% degree of deacetylation after 5 h of gelation at pH=5

Fig. 1 shows that the endothermic peak of deacetylated glucomannan appeared at a lower temperature than the native one. The endothermic peak shown in the DSC curve presents the water content in a sample. The acetyl group, responsible for glucomannan solubility, was removed during deacetylation that reduced the water-holding capacity of glucomannan. A similar result was explained by Li *et al.* (*31*), who found that the temperature of endothermic peak decreased with increasing DD of glucomannan. Deacetylation modified the exothermic peak of glucomannan between 300 and 600 °C. The unequal peaks between the native and the deacetylated one were an effect of the different reactions on breaking hydrogen bonds by increasing temperature (*29*). Guinesi and Cavalheiro (*32*) found that deacetylation moved the exothermic peak to a higher temperature due to the low acetyl content. Fig. 1 also demonstrates that the degradation of the deacetylated sample was observed at a lower temperature than that of the native one. This switched peak indicated that the thermal stability of the deacetylated glucomannan was weaker than of the native one. Wang *et al.* (*33*) suggested that the peak shift was due to the modification of crystallinity degree after deacetylation. Fewer calories were needed to complete the phase transition of the amorphous region since this structure was less compacted than that of the crystalline one (*33*).

### Size and appearance of fresh iron beads

The bead formation of deacetylated glucomannan occurred as there was a competition between the CaCl_2_ ions and glucomannan chains in water dissolution (*34*). Chen *et al.* (*35*) also explained that the high anionic charge of Cl^ˉ^ aggregated the glucomannan and induced the bead formation aside from making glucomannan less soluble.

**Fig. 2:**
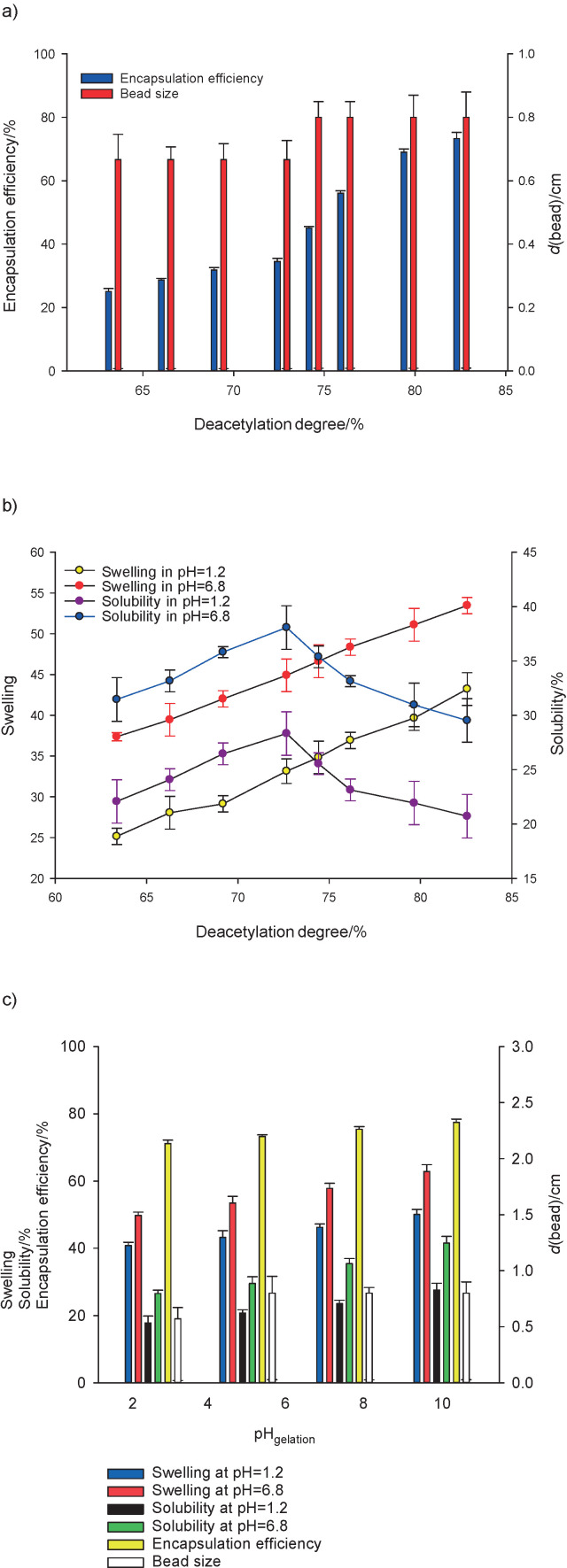
The effect of: a and b) glucomannan deacetylation degree, and c) gelation pH on encapsulation efficiency, bead size, swelling and solubility. The error bars show the data range obtained from triplicate experiments

The diameter size of the bead was not significantly affected by either the deacetylation degree or the pH of the gelling solution (Fig. 2). The average size of the bead was insignificantly different, ranging from 0.57 to 0.8 cm. Krasaekoopt and Bhandari (*36*) argued that the bead size and shape could be controlled by adjusting the dropping distance, needle diameter and properties of the matrix solution. In this study, none of the mentioned conditions was varied, except the property of the glucomannan used as matrix solution. Although deacetylation altered the glucomannan viscosity, the difference was not enough to make a significant variance of bead sizes (*20*, *33*).

**Fig. S1:**
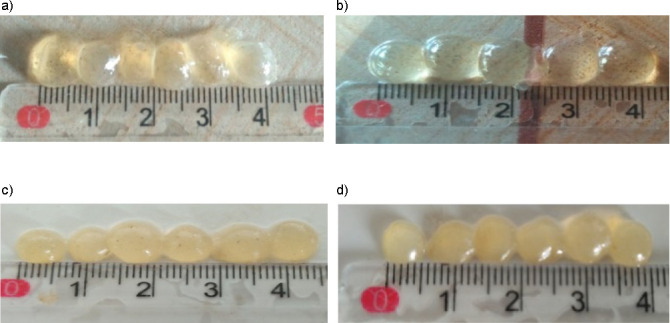
Visual appearance of iron beads for different degrees of deacetylation: a) 63.37, b) 69.19, c) 76.1 and d) 82.56%

Different viscosities of the matrix affected the shape of the beads (Fig. S1). The figures show that glucomannan with lower DD tended to form soft fragile oval round beads, whereas the glucomannan with higher DD formed more firm-tailed beads. This characteristic could be due to the improvement of gelling ability after deacetylation. Increasing DD modified glucomannan to be more hydrophobic, which resulted in faster gel formation after the deacetylated glucomannan solution was dropped to the CaCl_2_ solution for gelation. Moreover, deacetylation reduced the solubility of glucomannan, which promoted self-association between the molecules and thus enhanced gel strength (*37*). Enomoto-Rogers *et al.* (*38*) suggested that the mechanical properties were strongly affected by hydrogen bonding between hydroxyl groups, which could be controlled by the number of acetyl group substitutions. Ouyang *et al.* (*39*) reported similar results on the positive correlation between deacetylation and gel hardness.

Higher pH of the gelling solution produced murkier and more fragile beads (Fig. S1). Removing acetyl groups of glucomannan allowed it to aggregate through a linkage, such as a hydrogen bonding, which led to gel formation (*20*). However, the gel formation was also affected by the glucomannan concentration. Low glucomannan concentration (0.1 g/L) provides insufficient glucomannan molecules to form the aggregate, even under fully deacetylated conditions. The high viscosity of glucomannan can also cause the gelation to fail (*40*).

### Iron encapsulation efficiency

The ability of deacetylated glucomannan to entrap iron is represented by the encapsulation efficiency (Fig. 2a). The results revealed that encapsulation efficiency was positively correlated with DD. In this work, the highest encapsulation efficiency (73.27%) was observed at the highest DD (82.56%). The acetyl removal caused the aggregation of the glucomannan chains due to a reduction of a steric hindrance (*38*). This aggregation created by hydrogen bonding and hydrophobic interaction of deacetylated glucomannan molecules created a network. Moreover, higher DD also caused faster gelation and strengthened networking (*40*). As DD increased, crosslinks of deacetylated glucomannan molecules were more likely to occur, resulting in a more compact gel (*37*). Higher DD allowed the formation of more and bigger glucomannan networks, which provided more junctions to trap the active agents (*41*) and promoted adsorption of the agent on the surface (*37*).

The effect of pH of gelation on the encapsulation efficiency was less significant than that of the DD (Fig. 2b). Higher concentrations of hydroxyl groups were available at higher pH. Although this group contributed to acetyl replacement during deacetylation, the gelation process at higher pH was conducted at lower hydroxyl concentration than the deacetylation process. The remaining acetyl groups were at low concentration after deacetylation; hence, a significant further deacetylation reaction was less possible. As a result, insignificant improvement of encapsulation efficiency was observed with increasing solution pH.

### Swelling and solubility of iron encapsulation

Swelling and solubility of dried iron beads were carried out under two conditions: acidic and neutral. Solutions with pH=1.2 and pH=6.8 were selected to represent gastric and intestinal conditions, respectively, without the enzymes (*42*). Swelling is the migration of the liquid into the polymer matrix driven by osmotic pressure (*33*). Fig. 2 shows that swelling and solubility of the beads with variations of DD or pH of gelation were higher at pH=6.8 than those at pH=1.2. Higher pH increased the solubility and swelling of glucomannan because the low concentration of hydrogen ion in the solution does not affect the hydroxyl groups of glucomannan (*43*). The hydrogen ions from hydroxyl groups of glucomannan were transferred to the solution, which supported the glucomannan ionization and reaction with water molecules.

It has been reported that deacetylation reduces glucomannan solubility (*20*, *39*, *40*). However, the effect of DD on bead solubility was insignificant in both solutions. In this study, the beads were produced by dropping the deacetylated glucomannan into CaCl_2_ solution. The concentration of CaCl_2_ was comparable to the highest NaOH concentration used for the deacetylation. Since the bead samples were immersed for the same period of time, the interaction of the glucomannan with CaCl_2_ could modify the bead solubility. The result of this work supported the work of Kurt and Kahyaoglu (*40*), who found that the solubility of the beads was insignificantly different whether DD was 66 or 100%. In this work, the beads were created using 63.37–82.56% DD, which falls in the range of Kurt and Kahyaoglu’s report.

DD had a significant effect on the swelling of dried beads. Decreasing the number of acetyl groups reduced steric hindrance, which led deacetylated glucomannans to entangle and aggregate (*37*). This condition allowed water to enter between the glucomannans and swell accordingly.

The pH of gelation variations showed a significant positive effect on the solubility and swelling (Fig. 2c). Higher pH could lead to faster gelation, which indicated an increasing opportunity for the entanglement of macromolecular chains (*40*). During the immersion of the beads, the solvent plasticized stable forms of matrix molecules by bridging them through intermolecular hydrogen bonds (*31*). However, this bonding may not be the primary driving force for glucomannan gelation. It was suggested that both hydrogen bonding and hydrophobic interactions participated in the gelation and stabilization of glucomannan gels (*40*).

### Infrared spectra and morphological images

**Fig. 3:**
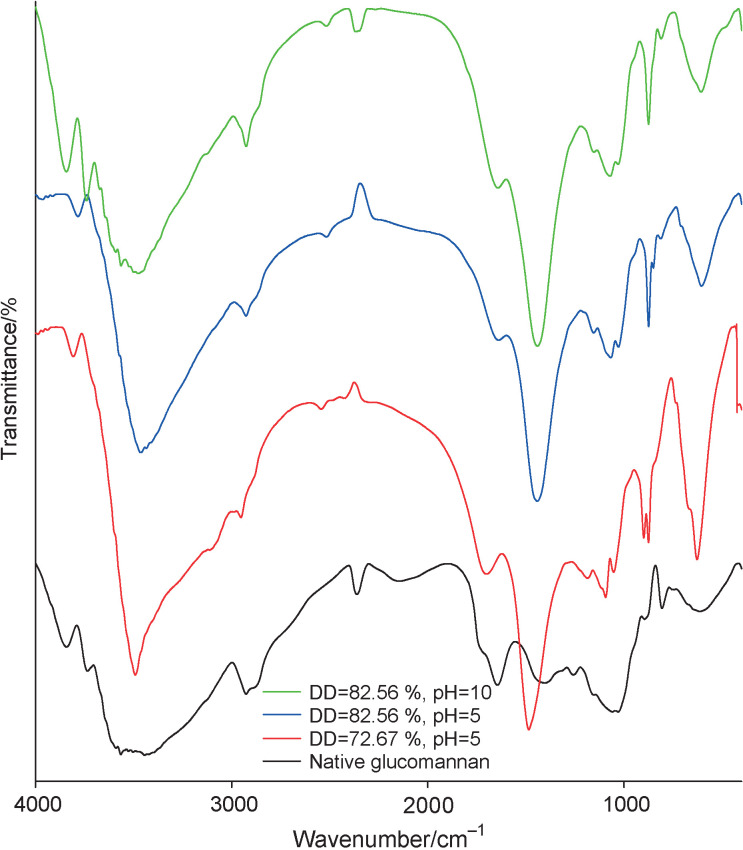
Infrared spectra of native and deacetylated glucomannan with various degrees of deacetylation (DD)

The infrared spectrum analysis was conducted to compare functional groups of native glucomannan and the encapsulated iron using different DD and gelation conditions to observe the functional group shift as a result of the deacetylation and gelation processes (Fig. 3). The functional groups of the native and the deacetylated glucomannan (DD=82.58 and 72.67%) which formed gel in the solution with the same pH were compared. Additionally, the results of gelation at different pH values (pH=10 and pH=5) were studied using the same deacetylated glucomannan. The hydroxyl group was detected in all samples as the broad peak at a wavenumber of approx. 3800 to 3000 cm^−1^. This band showed a reduction of transmittance of the iron bead samples at approx. 3450 cm^−1^, which was identified as hydroxyl group originating from crystal water in iron source (*44*). The bands at approx. 2900 and 1640 cm^−1^ were attributed to the vibration of C-H and C-O stretching of the hydroxyl group bound to water molecules (*37*). The absorbance of the stretching vibration peak, representing C-O of the acetyl group at approx. 1750 cm^−1^, was observed to decrease after deacetylation (*39*). This reduction indicated the success of acetyl replacement by alkaline during deacetylation. Moreover, the existence of C=O in COOH groups was identified at approx. 1460 cm^−1^. In general, there was insignificant change in the wavenumber among the samples. The results demonstrated that the backbone of glucomannan was not changed either by deacetylation or gelation pH. Ouyang *et al.* (*39*) also reported similar unchanged backbone.

**Fig. 4:**
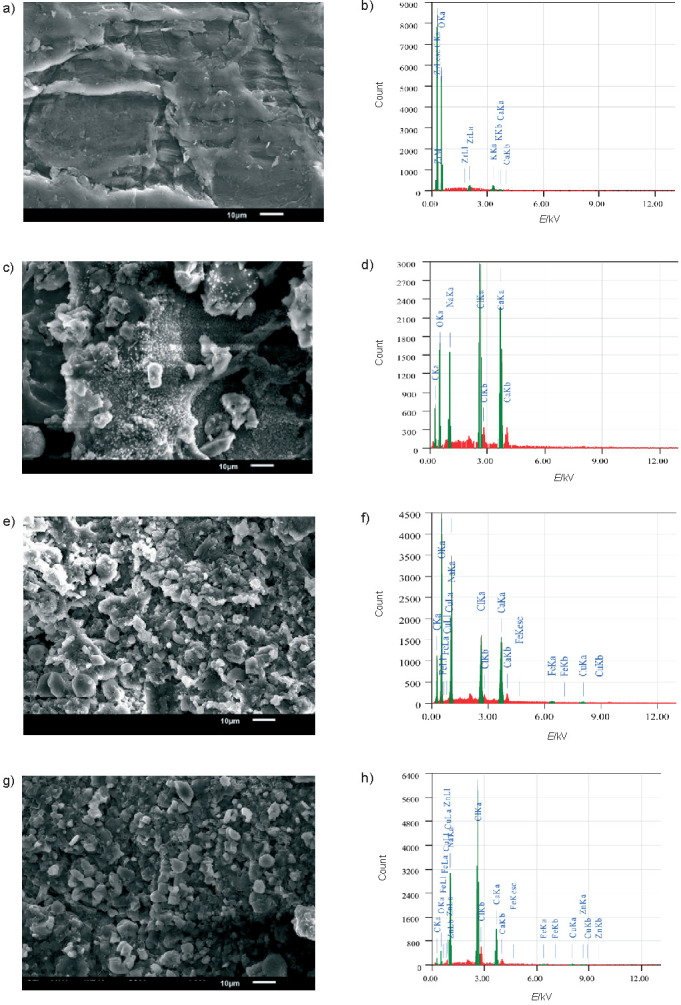
Morphology at 2500× magnification (left) and their energy-dispersive X-ray spectroscopy pattern (right) of: a and b) native glucomannan, c and d) deacetylated glucomannan at degree of deacetylation (DD)=82.56%, e and f) iron encapsulation using deacetylated glucomannan (DD=72.67%), and g and h) iron encapsulation using deacetylated glucomannan (DD=82.56%). The iron trace of the sample was shown by the code peak beginning with Fe

Fig. 4 shows on the left the surface appearance of native or deacetylated glucomannan and dried iron encapsulation using different DD. Deacetylation changed the smooth surface of glucomannan into rougher one. Ouyang *et al.* (*39*) reported a smoother and ordered structure of glucomannan after deacetylation. This opposite result could be due to different methods of deacetylation. These authors conducted deacetylation in ethanol, which resulted in a heterogeneous reaction (*39*). In this work, the deacetylation was homogenous, and required a drying process to obtain a deacetylated product. The iron addition resulted in more debris on the particle surface, which supported the proposed matrix-type encapsulation by deacetylated glucomannan. An insignificant difference in the particle surface appearance was observed for iron encapsulation using different DD. The energy-dispersive X-ray spectroscopy confirmed the entrapment of iron after the encapsulation (Fig. 4 on the right).

### Iron release kinetics

**Fig. 5:**
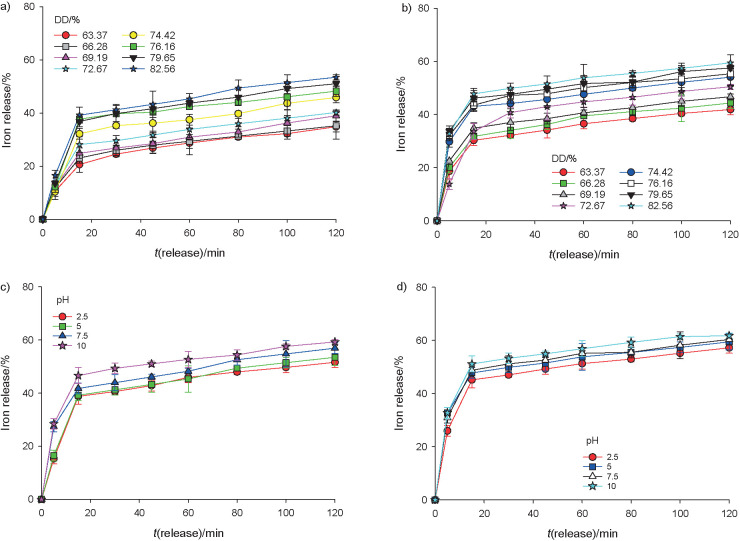
Profile of iron release from the matrix with various degrees of deacetylation (DD) in a solution at pH: a) 1.2 and b) 6.8, and gelation at pH: c) 1.2 and d) 6.8. The error bars show the data range obtained from triplicate experiments

The analysis of the release behaviour was conducted at two pH values, *i.e*. pH=1.2 and pH=6.8. The release profile shows that DD had a positive correlation with the iron release (Fig. 5). Glucomannan with higher DD entrapped more iron. However, the ability of the matrix to hold this higher concentration of iron was weakened when immersed in the solution due to a higher swelling capacity. The release of iron was higher at pH=6.8 than at pH=1.2 (Fig. 5). Both profiles showed two stages of release. A rapid release was observed in the first 15 min, followed by a lower rate of release. Goëlo *et al.* (*29*) suggested that the first stage of release was characterized by the cumulative concentration of a bioactive compound in the surrounding beads where the significant concentration difference was observed. The second stage occurred when different concentrations of the bioactive compound on the beads and in the released solution remained relatively constant due to lower release of iron. Different matrix encapsulation resulted in various controlled release profiles (*29*). This finding was supported by the swelling and solubility profile of the bead (Fig. 2). Increasing DD led to the improvement of the swelling degree of deacetylated glucomannan. The highest release of the iron was achieved at DD=82.56% at pH=1.2 and pH=6.8. A similar result was reported in previous findings by Wardhani *et al.* (*21*) and supported by the study of Wang *et al.* (*33*). Other than deacetylation process conditions, the iron release profile was also influenced by the pH of the gelation solution. Increasing the pH of the gelation improved the release ability of the glucomannan bead, which enabled the higher release of iron.

The iron encapsulated by deacetylated glucomannan with DD=63.37 and 82.56% was studied for their release profiles in pH=1.2 and pH=6.8 solution. These two samples were expected to show the effect of deacetylated glucomannan on the release profile. The release profile was described using Korsmeyer–Peppas, Weibull and Higuchi models. The Korsmeyer–Peppas model described the release of an active compound from a polymer matrix (*27*). The Weibull equation illustrated the dissolution occurrence, including the release profile of drugs from a matrix (*27*, *45*). The Higuchi model involves both dissolution and diffusion of a drug (*46*).

**Fig. 6:**
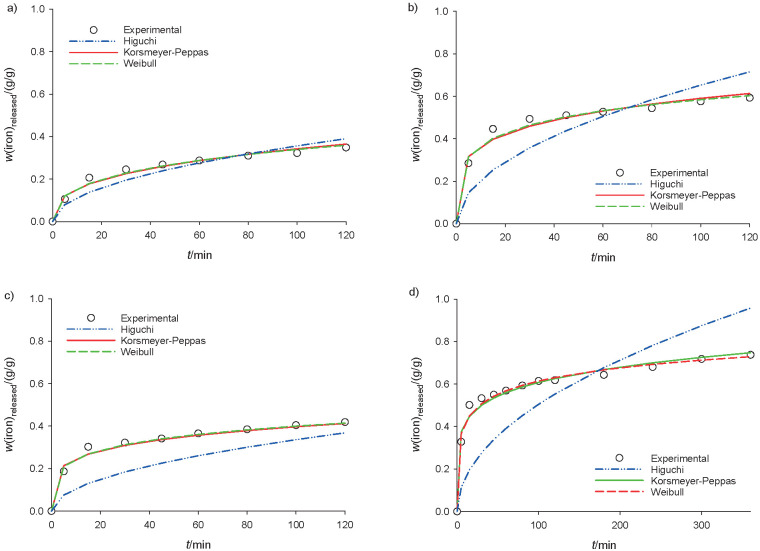
Plot of the mathematical models (Higuchi (*29*), Korsmeyer-Peppas (*27*) and Weibull (*28*)) of iron bead encapsulation with the lowest and highest encapsulation efficiency at: a and b) pH=1.2 and c and d) pH=6.8, respectively

The best-fitted model was confirmed by R^2^ and RMSE, shown together with other constants in Table 1 (*27*-*29*). Judging from both values, as well as the plotting profile (Fig. 6), the Weibull model was the best fit to describe the release profile of deacetylated glucomannan in the solutions at both investigated pH values, with R^2^≤0.93. The b<1 (0.257–0.391) of this model indicated that the release curve had a steep increase (*47*).

**Table 1:** Linear regression and constants of iron release models

DD/%	Release pH	Korsmeyer-Peppas (*27*)				Weibull (*28*)				Higuchi (*29*)		
n	a	R^2^	RMSE	a	b	R^2^	RMSE	K_h_	R^2^	RMSE
63.37	1.2	0.209	0.226	0.91	0.016	4.165	0.282	0.93	0.014	0.065	0.95	0.036
	6.8	0.161	0.289	0.92	0.016	3.254	0.246	0.95	0.014	0.051	0.907	0.105
85.22	1.2	0.346	0.070	0.95	0.025	14.578	0.391	0.96	0.022	0.036	0.98	0.107
	6.8	0.207	0.153	0.95	0.027	6.396	0.257	0.96	0.024	0.034	0.93	0.188

*DD=deacetylation degree

## CONCLUSIONS

Deacetylation modified glucomannan by changing its thermal properties Deacetylation and pH of gelation did not significantly influence the bead size but they affected the appearance of the fresh iron beads. These variables had a positive impact on the swelling of dried iron beads and improved the encapsulation efficiency of iron using the gelation method. The highest encapsulation efficiency (73.27%) was observed using 82.56% deacetylation degree. High swelling and encapsulation led to the release of more iron. The Weibull model was the best fit to represent the profile of iron release from deacetylated glucomannan using the gelation method (R^2^>0.93) in the solutions at both pH=1.2 and pH=6.8. The high encapsulation efficiency obtained with deacetylated glucomannan shows that deacetylation supports application of glucomannan as pH-sensitive matrix for iron encapsulation using gelation method.
